# Assessing the Role of Selenium in Endometrial Cancer Risk: A Mendelian Randomization Study

**DOI:** 10.3389/fonc.2019.00182

**Published:** 2019-03-27

**Authors:** Pik Fang Kho, Dylan M. Glubb, Deborah J. Thompson, Amanda B. Spurdle, Tracy A. O'Mara

**Affiliations:** ^1^Molecular Cancer Epidemiology Group, Genetics and Computational Biology Department, QIMR Berghofer Medical Research Institute, Brisbane, QLD, Australia; ^2^Department of Public Health and Primary Care, Centre for Cancer Genetic Epidemiology, University of Cambridge, Cambridge, United Kingdom

**Keywords:** Mendelian randomization, endometrial cancer, toenail selenium, circulating selenium, genome-wide association study

## Abstract

Endometrial cancer is the most commonly diagnosed gynecological cancer in developed countries. Based on evidence from observational studies which suggest selenium inhibits the development of several cancers (including lung and prostate cancer), selenium supplementation has been touted as a potential cancer preventative agent. However, randomized controlled trials have not reported benefit for selenium supplementation in reducing cancer risk. For endometrial cancer, limited observational studies have been conducted assessing whether selenium intake, or blood selenium levels, associated with reduced risk, and no randomized controlled trials have been conducted. We performed a two-sample Mendelian randomization analysis to examine the relationship between selenium levels (using a composite measure of blood and toenail selenium) and endometrial cancer risk, using summary statistics for four genetic variants associated with selenium levels at genome-wide significance levels (*P* < 5 × 10^−8^), from a study of 12,906 endometrial cancer cases and 108,979 controls, all of European ancestry. Inverse variance weighted (IVW) analysis indicated no evidence of a causal role for selenium levels in endometrial cancer development (OR per unit increase in selenium levels Z-score = 0.99, 95% CI = 0.87–1.14). Similar results were observed for sensitivity analyses robust to the presence of unknown pleiotropy (OR per unit increase in selenium levels Z-score = 0.98, 95% CI 0.89–1.08 for weighted median; OR per unit increase in selenium levels Z-score = 0.90, 95% CI = 0.53–1.50 for MR-Egger). In conclusion, these results do not support the use of selenium supplementation to prevent endometrial cancer.

## Introduction

Endometrial cancer is the most commonly diagnosed cancer of the female reproductive system in developed countries (1). Unlike breast and cervical cancers where a screening program is available to the general population, there is currently no available screening test for endometrial cancer and diagnosis relies on biopsy in symptomatic patients (2). Furthermore, the incidence of endometrial cancer is rising (3), highlighting the need for preventative measures. Selenium has received considerable attention as a possible cancer preventive agent [reviewed in (4)]. While randomized controlled trials have shown no benefit for selenium supplementation in reducing cancer risk over a period of up to 8 years (5), some observational longitudinal studies assessing selenium intake or selenium levels, over a period up to 25 years, have shown an inverse association between selenium and cancer risk [reviewed in (4)]. Thus, although findings from the longitudinal studies have been inconsistent (4), they may provide insight into the longer term effects of selenium exposure. A recent meta-analysis examining the association between selenium intake (dietary and supplemental) and overall cancer risk, has suggested that there was a reduction in cancer incidence among people consuming more than the recommended daily allowance of selenium (55 μg/day; RR = 0.96, 95% CI = 0.92–0.99) (6).

Very few studies have assessed the effects of selenium on endometrial cancer. In terms of cellular studies, it has been shown that a selenium metabolite can inhibit endometrial cancer cell proliferation, potentially through disruption of estrogen signaling (7). Findings from human studies, however, have been more equivocal. A population-based, case-control observational study of 417 endometrial cancer cases and 395 controls specifically assessed the role of dietary and supplemental selenium intake (as measured by questionnaire in the 6 months prior to diagnosis or enrolment as a control) in endometrial cancer development (8). In a comparison of the highest (≥103.2 μg) and lowest (<72.4 μg) selenium quartiles, this study did not support an association between selenium intake and endometrial cancer risk (OR = 0.74, 95% CI = 0.47–1.17) (8). Two small case-control studies (*n* < 100) have assessed serum selenium levels in endometrial cancer cases and controls. Sundstrom et al. (9) reported lower blood selenium levels in 64 cases as compared to 61 non-cancer controls, with an average of 1.01 ± 0.05 v 1.40 ± 0.08 μmol/L blood selenium in cases and controls, respectively (*P* < 0.001). A subsequent study of 35 endometrial cancer cases and 32 non-cancer controls reported a similar finding (average of 1.14 ± 0.04 vs. 1.26 ± 0.03 μmol/L blood selenium in cases and controls, respectively, *P* < 0.01) (10). Inconsistent results from these observational studies may be due to small sample sizes (8–10), reverse causation bias (9, 10), recall bias and measurement error in the dietary assessment (8). No prospective studies have examined the association of pre-diagnostic selenium levels with endometrial cancer risk. Thus, the role of selenium in endometrial cancer development remains inconclusive.

As no intervention study has yet been performed to explore the role of selenium in endometrial cancer risk, we employed a two-sample Mendelian randomization approach which uses germline genetic variants associated with selenium levels to proxy for selenium exposure (11). These germline genetic variants are largely independent from environment or lifestyle factors, and are established prior to disease onset, thus analyses using these genetic variants as instrumental variables are less susceptible to biases from confounding and reverse causation. Further, genetic effects on exposure of interest are lifelong, and hence it is comparable to a lifelong randomized controlled trial.

## Materials and Methods

Summary statistics for 12 genetic variants associated with selenium levels at genome-wide significance (*P* < 5 × 10^−8^) were extracted from a genome-wide association study (GWAS) meta-analysis of circulating selenium levels [*n* = 5,477; (12)] and toenail selenium levels [*n* = 4,162; (13)] in European-ancestry individuals. These variants were at two separate genetic loci; 5q14 (9 variants) and 21q22 (3 variants). To analyze the effect of selenium exposure on endometrial cancer risk, we used summary statistics from the Endometrial Cancer Association Consortium (ECAC) GWAS of 12,906 endometrial cancer cases and 108,979 controls of European descent (14). One of the 5q14 selenium-associated genetic variants, rs558133, was excluded because it was not assessed by the ECAC GWAS (it does not appear on the 1,000 Genomes v3 reference panel) and no proxy with *r*^2^ > 0.8 could be found. These potential instrumental variables were pruned for linkage disequilibrium (LD; *r*^2^ < 0.05) and four selenium-associated genetic variants (two independent variants per locus) remained as instrumental variables. We used PhenoScanner v2 (15) to explore the possibility of horizontal pleiotropy among the instrumental variables and their highly correlated variants (*r*^2^ > 0.8). Specifically, we examined traits associated with known risk factors of endometrial cancer (i.e., body mass index, age at menarche, age at menopause, postmenopausal serum estradiol levels, nulliparity, infertility, and insulin levels) in the published literature at *P* < 7.14 × 10^−3^ (i.e., 0.05/number of known risk factors explored, *n* = 7); none of these instrumental variables were associated with these traits.

The reported effect for circulating and toenail selenium instrumental variables was expressed in Z-score units per effect allele. For the purpose of Mendelian randomization analysis, Z-scores were converted to beta and standard error values using the following equations, as per Taylor et al. (16), where N is the sample size, eaf is the effect allele frequency, and SE is the standard error of converted beta:

$$\begin{array}{l} Beta=\;\frac{Z-score}{\sqrt{N}}\times \frac{1}{\sqrt{eaf\left(1-eaf\right)}}\\ SE=\frac{Beta}{Z-score} \end{array}$$

Converted selenium level summary statistics for these instrumental variables and their association with endometrial cancer risk are shown in Table 1. Because summary statistics were expressed in Z-scores, neither the converted beta values for associations of genetic variants with selenium levels nor the effect sizes from the Mendelian randomization analysis have interpretable units, however they do provide the direction and statistical strength of associations.

**Table 1:** Genetic associations with selenium levels and endometrial cancer risk.

**Instrumental variables**	Chr:Pos^*^	**R^**2**^^†^**	**EA**	**OA**	**EAF_**Se**_**	***Z*-score**	**Beta_**Se**_**	**SE_**Se**_**	***P*_**Se**_**	**EAF_**EC**_**	**Beta_**EC**_**	**SE_**EC**_**	***P*_**EC**_**
rs1789953	chr21:44482936	0.04	T	C	0.14	5.52	0.16	0.03	3.4 × 10^−8^	0.13	−0.04	0.02	0.12
rs6586282	chr21:44478497		T	C	0.17	−5.89	−0.16	0.03	3.96 × 10^−9^	0.17	−0.04	0.02	0.04
rs6859667	chr5:78745042	0.03	T	C	0.96	−6.92	−0.36	0.05	4.4 × 10^−12^	0.96	0.02	0.04	0.54
rs921943	chr5:78316476		T	C	0.29	13.14	0.29	0.02	1.9 × 10^−39^	0.29	0.00	0.02	0.90

* *from hg19*;

† *pairwise LD in Europeans (1000 Genomes) provided for instrumental variables at the same locus*;

*Se, Selenium; EC, Endometrial cancer; EA, Effect allele; OA, Other allele; EAF, Effect allele frequency from each GWAS; Beta, effect size; SE, Standard error; P, P-value. Beta_EC_ and SE_EC_ are the natural log odds ratio of endometrial cancer risk and associated standard error, respectively. Estimates for Selenium levels have been taken from (13) and estimates for EC from (14)*.

Individual Wald-type ratios for each of the instrumental variables were determined as a ratio of instrumental variable-endometrial cancer regression over the instrumental variable-selenium levels regression (17). Individual Wald-type ratios were meta-analyzed using the inverse variance weighted (IVW) approach. A random effect model was used to account for heterogeneity. The IVW approach assumes that instrumental variables do not exhibit horizontal pleiotropy (where a single genetic variant has simultaneous effects on other phenotypes that affect the outcome independently of the exposure of interest) or, if this is violated, that the horizontal pleiotropy is “balanced” across all instrumental variables. Thus, we implemented sensitivity analyses that are more robust to pleiotropy when it is “unbalanced” (i.e., exhibiting directional pleiotropy): (i) weighted median analysis, which provides valid causal estimate even when up to 50% of the weight comes from instrumental variables with horizontal pleiotropic effects (18); and (ii) random effect MR-Egger analysis, which provides valid pleiotropy-corrected causal estimates even if all instrumental variables are invalid (19). MR-Egger analysis corrects for the directional pleiotropy by introducing an intercept which captures the average pleiotropic effects of all included variants on the outcome. An exponentiated MR-Egger intercept that deviates from 1 is an indicator of directional pleiotropy. It should also be noted that the validity of IVW and MR-Egger regression estimates rely on satisfaction of the InSIDE (instrument strength independent of direct effect) assumption where the instrument strength does not correlate with the horizontal pleiotropic effects on the outcome (19).

To assess the strength of the instruments, F statistics and the proportion of variance (R^2^) in circulating and toenail selenium explained by instrumental variables were calculated as per Rees et al. (20) and Yarmolinsky et al. (21). We used the $I_{GX}^{2}$ (22) statistic to assess weak instrument bias for MR-Egger analysis using the “MendelianRandomization” package in R (23). This statistic quantifies the regression dilution bias due to violation of the NO Measurement Error (NOME; genetic associations with exposure of interest are measured without error) assumption. An $I_{GX}^{2}$ statistic approaching 1 indicates that violation of the NOME assumption does not substantially dilute the effect estimates of MR-Egger analysis toward a null association. Unless otherwise stated, Mendelian randomization analyses were performed using the “TwoSampleMR” package in R (24).

## Results

The combined multi-allelic instrument explained 2.9% of the variation in circulating and toenail selenium levels. Individual Wald-type ratios and F statistics for instrumental variables are presented in Table 2. F statistics for these instrumental variables were all >10 (range 19.24–44.55) indicating instruments were unlikely to suffer from weak instrument bias. Mendelian randomization analysis did not support an association between selenium levels and endometrial cancer risk using the IVW method (OR per unit increase in selenium levels Z-score = 0.99, 95% CI = 0.87–1.14, *P* = 0.93). We found limited evidence for heterogeneity amongst the individual causal estimates for the included variants by Cochran's Q statistic (25) (Cochrain's Q statistics = 7.22, *P* = 0.07). The exponentiated intercept of MR-Egger regression was 1.03 (95% CI = 0.91–1.16, *P* = 0.72) and therefore provided no evidence of directional pleiotropy across the multi-allelic instrument. Further, the $I_{GX}^{2}$ statistic, quantifying weak instrument bias in the context of MR-Egger, was minimal ($I_{GX}^{2}$= 92%). This suggests that any potential bias toward a null association as a result of NOME violation is ≤8%. Association estimates from sensitivity analyses (MR-Egger regression and weighted median analysis) were consistent with that reported by IVW analysis (OR per unit increase in selenium levels Z-score = 0.90, 95% CI = 0.53–1.50, *P* = 0.72 for MR-Egger; OR per unit increase in selenium levels Z-score = 0.98, 95% CI = 0.89–1.08, *P* = 0.70 for weighted median).

**Table 2:** F statistics and Individual Wald-type ratios for all instrumental variables.

**Instrumental variables**	**F statistic**	**Beta_**Se-EC**_**	**SE_**Se-EC**_**	***P*_**Se-EC**_**
rs1789953	34.07	−0.22	0.14	0.12
rs6586282	36.88	0.26	0.13	0.04
rs6859667	19.24	−0.07	0.11	0.54
rs921943	44.55	−0.01	0.06	0.89

*Se, Selenium; EC, Endometrial cancer; Beta, effect size in standard deviation unit; SE, Standard error; P, P value*.

## Discussion

To our knowledge, this is the first Mendelian randomization study evaluating the effect of selenium on endometrial cancer. This analysis does not support a causal relationship between selenium levels and endometrial cancer risk. However, given the fact that the combined multi-allelic instrument explains a small amount of the variance in circulating and toenail selenium levels (<3%), the power to detect a causal association in Mendelian randomization analysis may be limited and thus, we cannot rule out the possibility that genetically predicted selenium levels have some effect on endometrial cancer risk. This analysis should be revisited when more genome-wide significant selenium variants are identified from future, larger GWAS studies. Further, statistical power for Mendelian randomization analyses may also be increased through the use of more precise effect estimates from larger GWAS of endometrial cancer.

The validity of Mendelian randomization analysis holds under the condition that three important assumptions are fulfilled. These assumptions require that genetic variants chosen as instrumental variables are:

1. Strongly associated with the exposure of interest
2. Not associated with any confounder(s) that affects the relationship between the exposure of interest and outcome
3. Not associated with outcome, independent of the exposure (i.e., no horizontal pleiotropy).

Our instrumental variables have high F-statistics (>10), thus fulfilling assumption 1. Assumptions 2 and 3 are difficult to validate. We have attempted to minimize violation of assumption 2 by scanning associations of instrumental variables from the literature, finding none of the instrumental variables to be associated with known endometrial cancer risk factors. However, we are limited in exploring this assumption by the GWAS that have been conducted for these risk factors, and we cannot discount the possibility that associations between these variants and unknown endometrial cancer risk factors may exist. Sensitivity testing (by MR-Egger regression and weighted median analysis) has been used to address assumption 3 and we have not found evidence that this assumption has been violated. However, given the limitations of these tests (e.g., the low statistical power of the MR-Egger intercept test, discussed below), we cannot rule out this possibility.

The strengths of our study include incorporation of multiple selenium level-associated genetic variants as a multi-allelic instrument to maximize the variation in selenium levels explained; and use of the largest available GWAS datasets to provide the greatest statistical power possible. Limitations of this study include use of instrumental variables from mixed gender GWAS which were assessed in female-only endometrial cancer GWAS. Although both selenium GWASs controlled for the effect of sex, we cannot not exclude the possibility that there is a residual effect of this covariate which may violate the assumption that instrumental variables are strongly associated with the exposure. A potential limitation of two-sample Mendelian randomization is that by using two different GWAS sample sets to obtain the instrumental variable-exposure and -outcome effect, population stratification may have confounded the observed associations despite all populations being of European descent. Weaknesses of the MR-Egger regression sensitivity analysis performed in our study include its relatively lower statistical power as compared to the IVW and weighted median analysis methods, and its vulnerability to weak instrument bias which may bias MR-Egger regression toward the null (19). However, we assessed the extent to which weak instrument bias may have affected our MR-Egger results using the $I_{GX}^{2}$ statistic, and found it to be negligible.

The identification of preventative agents for cancer is an attractive avenue of research because unlike other approaches for disease prevention, such as lifestyle changes, taking a dietary supplement (e.g., selenium) should be considerably easier to implement. Candidate dietary supplements can be identified by observational studies; however, moving these candidates through to human use requires the establishment of expensive randomized controlled trials. For example, a recent prostate cancer prevention trial, examining the benefit of selenium and/or vitamin E supplement on cancer risk, failed because of adverse effects and lack of efficacy, at a cost of >US$110 million (26, 27); whereas, a subsequent Mendelian randomization study was able to recapitulate the results of this trial using publicly available GWAS data (21).

In conclusion, Mendelian randomization analysis provided no support for selenium supplementation in the prevention of endometrial cancer. More generally, these findings further highlight the value of Mendelian randomization for rapidly excluding proposed interventions that are unlikely to be successful, prior to the initiation of expensive and lengthy trials. This approach could allow resources to be targeted toward trials of alternative interventions with more promising genetic evidence.

## Data Availability

The datasets for this manuscript are not publicly available because Data are available from the authors of the original papers on request. Requests to access the datasets should be directed to Tracy O'Mara, tracy.omara@qimrberghofer.edu.au.

## Author Contributions

PK, DG, DT, AS, and TO conception or design of the work; the acquisition, analysis and interpretation of data for the work, drafting the manuscript, and agreement to be accountable for all aspects of the work in ensuring that questions related to the accuracy or integrity of any part of the work are appropriately investigated and resolved. All authors were involved in revision of the manuscript and provide final approval of the version to be published.

### Conflict of Interest Statement

The authors declare that the research was conducted in the absence of any commercial or financial relationships that could be construed as a potential conflict of interest.
